# Endogenous endophthalmitis: A case of a presumed mixed intraocular opportunistic infection by a fungal species and cytomegalovirus

**Published:** 2019

**Authors:** Athanassios Giarmoukakis, Styliani Blazaki, Aikaterini Chalkia, Argyro Plaka, Georgios Bontzos, Miltiadis Tsilimbaris

**Affiliations:** * Department of Ophthalmology, University of Crete, Heraklion, Greece

**Keywords:** endogenous fungal endophthalmitis, cytomegalovirus, mixed intraocular infection, opportunistic intraocular infection

## Abstract

Background: Endogenous endophthalmitis is a serious sight-threatening disease. Common causes include immunocompromised state and intravenous drug use, permitting opportunistic pathogens to reach the eye through the blood stream. We reported a rare case of a presumed simultaneous opportunistic intraocular fungal and cytomegalovirus (CMV) infection.

**Case presentation:** A 67-year-old male patient with a recent history of hospitalization due to pneumonia, presented to our department with bilateral loss of vision. Ocular examination revealed low visual acuity, signs of vitritis with chorioretinal infiltrations and cotton ball colony-like lesions, bilaterally. A bilateral endogenous fungal endophthalmitis was suspected and topical and systemic antifungal treatment was initiated. Nevertheless, vitreous and blood cultures were negative for fungi and other bacteria, while serological examinations revealed primary infection with CMV. Following vitrectomy, polymerase chain reaction (PCR) of vitreous washings confirmed the intraocular infection with CMV. Treatment was modified, including intravenous administration of Gancyclovir. In the following days, the patient’s clinical signs and visual acuity improved remarkably.

**Conclusions:** A case of a presumed mixed fungal and CMV intraocular infection was presented. High level of suspicion with prompt diagnosis and aggressive combination treatment led to a favorable result.

## Introduction

Endogenous endophthalmitis is a vision threatening intraocular inflammation caused by the systemic hematogenous spread of infectious pathogens that cross the blood-ocular barrier, inoculating mainly the choroid and/ or the retina [**1**]. It is considered a relatively uncommon intraocular infection, accounting for 2% to 16% of all endophthalmitis cases [**2**,**3**]. In contrast with other types of endophthalmitis, the most common causative pathogens are usually fungal species [**4**,**5**], with Candida albicans followed by Aspergillus species being the most commonly isolated microorganisms [**4**,**5**].

Cytomegalovirus (CMV) retinitis is the most common opportunistic intraocular infection in immunocompromised patients, including patients with acquired immunodeficiency syndrome (AIDS) [**6**]. Despite the strong association of the disease with immunosuppression, cases of CMV retinitis in immunocompetent individuals have been described in literature, with most of them manifesting in diabetic patients after intravitreal steroid administration [**7**,**8**]. Prompt diagnosis and treatment of the disease are considered essential for the preservation of vision.

Within this context, we reported a rare case of a presumed mixed fungal and CMV intraocular infection.

## Case Presentation

A 67-year-old male presented to our department complaining for bilateral blurred vision of one-month duration, starting during a recent two-month hospitalization at a different hospital for severe acute pneumonia, which included a one-week admission to the intensive care unit. The patient was discharged two weeks prior to his presentation with an antibiotic and corticosteroid (methylprednisolone) oral regimen. His past medical history was remarkable for coronary artery disease with a history of coronary artery bypass surgery, systemic hypertension, and hyperlipidemia. 

**Fig. 1:**
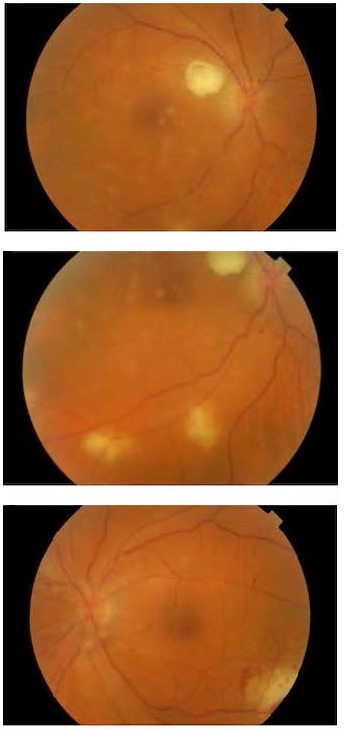
Fundus images at presentation. Bilateral vitreous haze, chorioretinal lesions, and presence of a large “cotton-ball” colony in the patient’s right eye

At presentation, his best-corrected visual acuity (BCVA) was 20/80 in his right eye and 20/ 63 in his left eye. Slit-lamp biomicroscopy revealed an anterior chamber reaction of 2+ and 1+ in his right and left eye, respectively. Vitreous haze was graded 1+/ 2+ and 1+ in his right and left eye, respectively. Fundoscopy revealed several small whitish pre-retinal lesions within the posterior pole, as well as larger whitish-yellow chorioretinal lesions within the retinal vascular arcades with associated perilesional subretinal hemorrhages, bilaterally (**Fig. 1**). Moreover, a large pre-retinal whitish round lesion temporarily to the optic nerve was evident in the patient’s right eye, resembling a large “cotton-ball” colony (**Fig. 1**). 

A bilateral endogenous fungal endophthalmitis was strongly suspected and a pars-plana vitreous aspiration needle tap was performed in his right eye after admission, followed by an intravitreal injection of Voriconazole 0.2mg/ 0.1ml. Vitreous aspiration samples were sent to the Microbiology Laboratory for cultures and stains. In addition, systemic steroid uptake was discontinued, and systemic antifungal treatment with oral Voriconazole 200mg b.i.d was initiated, and the patient underwent a complete lab examination, including serological tests for a series of infectious pathogens, both bacterial and viral, as well as blood and urine cultures. An intravitreal (IVT) injection of Voriconazole 0.2mg/ 0.1ml was repeated bilaterally two days later as a relative deterioration of his clinical picture was noted.

**Fig. 2:**
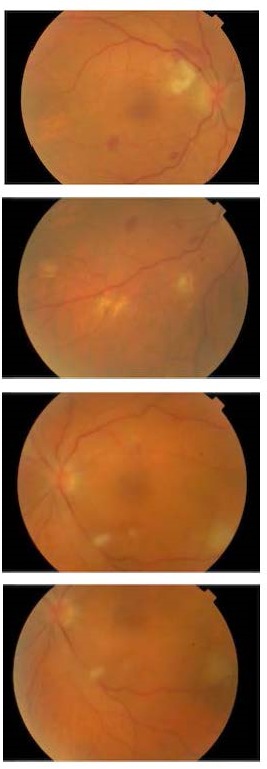
Clinical picture on eighteenth day, following combination systemic and topical treatment bilaterally, combined with vitrectomy in the patient’s right eye. Significant improvement of chorioretinal lesions and vitreous haze is noted

Five days post-admission vitreous cultures and stains were negative for microorganisms, while his serological tests indicated a primary cytomegalovirus (CMV) infection with positive IgM antibody titers for CMV. Apart from this finding, his rest lab exams were unremarkable, including blood and urine cultures, while the patient remained afebrile. Meanwhile, the patient’s fundoscopic picture continued to deteriorate with a mild increase in vitreous haze and the appearance of new intra-retinal hemorrhages. Therefore, on the sixth day, a vitrectomy was performed in his right eye for both therapeutic and diagnostic purposes, accompanied by an IVT administration of Amphotericin B 5μg/ 0.1ml. Two days following vitrectomy, cultures and stains of vitreous washings were again negative, while the presence of CMV DNA in the vitreous was confirmed via polymerase chain reaction (PCR). Intravenous (IV) Gancyclovir 225mg b.i.d was initiated, accompanied by bilateral IVT administration of Amphotericin B 5μg/ 0.1ml every second day (four IVT injections in each eye in total). Administration of Voriconazole 200mg b.i.d orally was continued. In the following days, significant improvement was recorded with gradual regression of fundus lesions and improvement of the patient’s BCVA (**Fig. 2**).

**Fig. 3:**
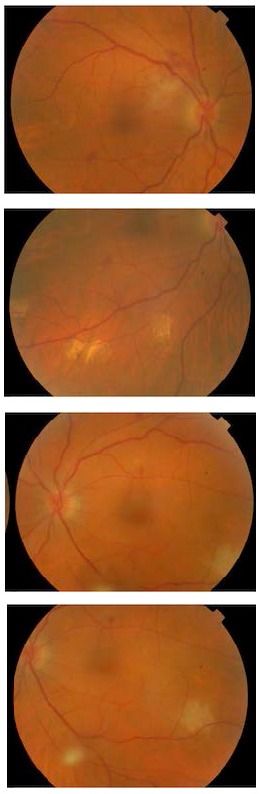
Fundus images one and a half month post-admission with almost complete resolution of clinical lesions

The patient was discharged on the eighteenth day with a maintenance oral regimen of Voriconazole 200mg b.i.d and Valganciclovir 450mg daily. During his follow up visit, almost three weeks later, his BCVA had improved to 20/ 40 bilaterally, chorioretinal lesions had completely resolved leaving well demarcated areas of chorioretinal atrophy and vitreous was relatively clear (**Fig. 3**).

Endogenous endophthalmitis has been strongly associated with a series of predisposing risk factors, that include, among others, immunosuppression, immunosuppressive treatment, malignancies, intravenous drug abuse, catheters, parenteral nutrition, and urinary and pulmonary tract infections [**4**,**9**]. Nevertheless, Shankar et al. [**10**] recently reported a case series of culture proven endogenous bacterial endophthalmitis in otherwise healthy individuals. Endogenous fungal endophthalmitis is the dominant variant of the disease [**4**,**5**]. On the other hand, intraocular infection by CMV is a well-documented condition within immunocompromised patients [**6**]. Cases of CMV retinitis have been reported following periocular or intraocular corticosteroid administration [**7**,**8**], as well as in immunocompetent individuals without any identifiable risk comorbidities such as drug induced immunosuppression or diabetes mellitus [**11**,**12**]. Moreover, in a recent article of Downes et al. [**13**], systemic use of corticosteroids was one of the most identifiable risk factors reported in cases of CMV retinitis and infection in patients negative for human immunodeficiency virus (HIV) infection, other immunodeficiency syndromes or prior periocular or intraocular steroid administration. 

In our case, the patient’s clinical picture upon presentation was compatible with endogenous fungal endophthalmitis. The presumptive diagnosis was further supported by the patient’s recent two-month hospitalization due to acute pneumonia and long-term systemic use of corticosteroids. Despite our strong clinical suspicion, we were unable to confirm our presumptive diagnosis despite repeated vitreous cultures, while we encountered the unexpected finding of positive serological indication of primary CMV infection. At the same time, the patient’s clinical picture continued to deteriorate, despite systemic and intravitreal administration of antifungal agents, and a vitrectomy in his right eye was decided. Following vitrectomy, PCR confirmed the presence of CMV viral load in the patient’s vitreous, while new vitreous cultures remained negative for both bacterial and fungal pathogens. Despite the poor clinical evidence of CMV-related retinitis, possibly excluding the appearance of new dot intra-retinal hemorrhages, we decided to change our therapeutic approach, including both antiviral and antifungal systemic treatment and IVT administration of Amphotericin B instead of Voriconazole. The aforementioned approach led to significant regression of clinical lesions, up to almost complete resolution on the patient’s first follow up visit, while continuing both antifungal and antiviral maintenance therapy.

Weiss et al. [**14**] reported a case of simultaneous aspergillus endogenous endophthalmitis and CMV retinitis following kidney transplantation. To our knowledge, this is the second reported case of a mixed fungal and CMV intraocular infection. A limitation of our report is that no fungal species was isolated from cultures to confirm our diagnosis. Nevertheless, high culture negativity rates in cases of endogenous endophthalmitis have been previously documented in large case series [**9**]. On the other hand, while CMV intraocular infection was confirmed by PCR, poor clinical evidence of typical CMV infection, such as retinitis or vasculitis, could be documented. A presumptive early-subclinical stage of the indolent type of the viral insult combined with the prompt commencement of antiviral treatment after serological evidence of acute CMV infection and the discontinuation of systemic steroids could potentially explain the lack of typical clinical signs in our case. Whether the systemic and topical antifungal treatment alone combined with the therapeutic vitrectomy performed in the patient’s right eye would have led to the same favorable result remains unknown to the authors.

## Conclusions

Within this context, authors documented a rare case of a presumed mixed intraocular opportunistic infection by a fungal species and CMV in a patient with a recent history of a long-term hospitalization and systemic corticosteroid uptake. The combination of both systemic and topical antifungal and antiviral treatment with surgical intervention led to a successful therapeutic outcome. 

**Acknowledgments**

None.

**Sources of funding**

None.

**Disclosures**

None.
